# Utility of a Sequence-Independent, Single-Primer-Amplification (SISPA) and Nanopore Sequencing Approach for Detection and Characterization of Tick-Borne Viral Pathogens

**DOI:** 10.3390/v13020203

**Published:** 2021-01-29

**Authors:** Annika Brinkmann, Steven Uddin, Eva Krause, Rebecca Surtees, Ender Dinçer, Sırrı Kar, Sabri Hacıoğlu, Aykut Özkul, Koray Ergünay, Andreas Nitsche

**Affiliations:** 1 Center for Biological Threats and Special Pathogens 1 (ZBS-1), Robert Koch Institute, 13353 Berlin, Germany; BrinkmannA@rki.de (A.B.); uddinstv@rki.de (S.U.); krausee@rki.de (E.K.); surteesr@rki.de (R.S.); NitscheA@rki.de (A.N.); 2 Department of Virology, Faculty of Veterinary Medicine, Dokuz Eylül University, İzmir 35890, Turkey; enderdin@gmail.com; 3 Department of Biology, Namık Kemal University, Tekirdağ 33110, Turkey; sirrikar@yahoo.com; 4 Department of Virology, Faculty of Veterinary Medicine, Ankara University, Ankara 06110, Turkey; saabrii@hotmail.com (S.H.); ozkul@ankara.edu.tr (A.Ö.); 5 Virology Unit, Department of Medical Microbiology, Faculty of Medicine, Hacettepe University, Ankara 06110, Turkey

**Keywords:** NGS, SISPA, crimean-congo hemorrhagic fever, jingmen tick virus, tick

## Abstract

Currently, next generation sequencing (NGS) is the mainly used approach for identification and monitorization of viruses with a potential public health threat in clinical and environmental samples. To facilitate detection in NGS, the sequence-independent, single-primer-amplification (SISPA) is an effective tool for enriching virus sequences. We performed a preliminary assessment of SISPA-nanopore sequencing as a potential approach for screening tick-borne viruses in six specimens with detectable Crimean-Congo hemorrhagic fever virus (CCHFV) and Jingmen tick virus (JMTV) sequences. A comparison of unbiased NGS and SISPA followed by nanopore sequencing was carried out in 4 specimens with single and pooled ticks. The approach was further used for genome sequencing in culture-grown viruses. Overall, total/virus-specific read counts were significantly elevated in cell culture supernatants in comparison to single or pooled ticks. Virus genomes could be successfully characterized by SISPA with identities over 99%. Genome coverage varied according to the segment and total read count. Base calling errors were mainly observed in tick specimens and more frequent in lower viral loads. Culture-grown viruses were phylogenetically-related to previously-reported local viruses. In conclusion, the SISPA + nanopore sequencing was successful in generating data comparable to NGS and will provide an effective tool for broad-range virus detection in ticks.

## 1. Introduction

In the current global environment struck by the ongoing pandemic from a novel virus, identification of agents with a potential public health threat have never gained more attention. Widespread availability of massively parallel sequencing platforms and related protocols (frequently called next generation sequencing, NGS) have enabled metagenomic investigation on viruses and facilitated the discovery of several novel agents [1]. Moreover, NGS-based approaches such as nanopore sequencing can also be used for accurate identification of etiologic agents, monitorization and molecular epidemiology analyses during outbreaks in real time [2]. However, these techniques are frequently hampered by the relatively lower abundance of virus genomes within a huge sequence background from other cellular sources. To overcome this problem, several approaches for enriching the viral nucleic acid content in the specimen have been developed [3]. Based on random priming and nonspecific amplification, the sequence-independent, single-primer-amplification (SISPA) is a frequently employed method for this purpose. Combined with nuclease treatment and subsequent NGS, it enables metagenomic identification of viruses, surpassing the capacity of any type-specific or broad-range polymerase chain reaction (PCR)-based detection [4,5,6]. 

Viruses transmitted by ticks comprise a large group in vector-borne viruses. Crimean-Congo hemorrhagic fever virus (CCHFV) is the causative agent and the most notable tick-borne infection due to its widespread distribution and health impact [7]. Lacking an effective specific treatment or vaccine, CCHFV is among the top agents in the World Health Organization’s blueprint list of priority diseases [8]. The CCHFV genome comprises single-stranded RNA in three segments, encoding for the nucleocapsid (S segment), envelope glycoproteins (M segment) and viral polymerase (L segment) [9]. Jingmen tick virus (JMTV) is a recently identified tick-borne virus with confirmed human infections [10]. JMTV exhibits a unique genome configuration, comprising four positive-sense RNA segments where two segments encoding for nonstructural proteins are related to flavivirus proteins [11]. Current information on JMTV and related viruses is limited; they appear as globally spread viruses capable of infecting humans and various arthropods or animals [12].

The impact of SISPA-based enrichment and nanopore sequencing has not yet been investigated for vector-borne viruses transmitted by ticks. This study was carried out as a preliminary effort to assess the impact of this approach for screening particular tick-borne viral pathogens.

## 2. Materials and Methods

### 2.1. Specimens, NGS and SISPA

The tick specimens were pooled according to species and collection site up to a maximum of 50 individuals, ground by vortexing with tungsten carbide or stainless-steel beads (Qiagen, Hilden, Germany), in 500–700 μL of Eagle’s minimal essential medium, supplemented with 5% fetal bovine serum and 1% L-glutamine and. Each pool was subsequently centrifuged at 4000 rpm for 4 min, and the supernatant was aliquoted and stored at −80 °C. In addition to the tick pools, African green monkey kidney (Vero E6, ATCC: CRL-1586) cell culture supernatants with detectable target viruses on the first passage were evaluated. Fresh RNA from processed tick specimens and cell cultures supernatants were prepared using QIAamp viral RNA kit (Qiagen, Hilden, Germany) without carrier RNA in an individual elution volume of 60 µL. For Illumina sequencing without previous SISPA, cDNA and double strand cDNA synthesis was performed using Superscript IV reverse transcriptase (Thermo Fisher Scientific, Darmstadt, Germany) and Nondirectional RNA Second Strand Synthesis Module (New England Biolabs, Ipswich, MA, USA), The sequencing runs on the tick pools were carried out as described previously, using Illumina instruments (Illumina, San Diego, CA, USA) [13,14]. The SISPA prior to nanopore sequencing was carried out according to a protocol described previously [15]. In brief, following DNAse treatment, SISPA primers A and B were employed for first strand and second strand synthesis/cDNA amplification, respectively; using the Invitrogen SuperScript IV First-Strand Synthesis System (Thermo Fisher Scientific). The products were cleaned up using the Agencourt AMPure XP reagent (Beckman Coulter Biosciences, Krefeld, Germany) and quantitated using Qubit dsDNA HS Assay Kit (Thermo Fisher Scientific). Individual specimen sequencing libraries were prepared using the Ligation Sequencing Kit (SQK-LSK109) (Oxford Nanopore Technologies, Oxford, UK). For combined sequencing of several samples on one flow cell, samples were barcoded with the Native Barcoding Expansion Kit (EXP-NBD104 and EXP-NBD114). The libraries were sequenced for 3 h on Oxford Nanopore MinION SpotON Flow Cells Mk I, R9.4 (Oxford Nanopore Technologies).

The Fast5 sequences generated during sequencing were transcribed to FastQ sequences by using Guppy v.3.4.5 (Oxford Nanopore Technologies) on the MinION IT device (MNT-001). Computational separation of the barcoded samples was performed with Guppy v.3.4.5 for Windows with standard parameters. Adapter and primer sequences were removed by using Guppy v.3.4.5 and Geneious Prime 2.1 (Biomatters Ltd., Auckland, New Zealand). The trimmed FastQ sequences for each sample were aligned to the corresponding virus reference if available with Guppy v.4.0.11 for Linux, and the resulting alignments were used for calculation of accuracy and genome coverage of the consensus sequence.

### 2.2. Sequence Handling and Phylogenetic Analysis

Consensus sequences were handled using Geneious (Biomatters Ltd.). BLASTn and MEGABLAST algorithms were used for nucleotide similarity searches in the National Center for Biotechnology Information (NCBI) database [16]. Alignment and pairwise sequence comparisons were carried out using CLUSTAL W [17]. Evolutionary history was inferred via the maximum-likelihood method, using the substitution model estimated as optimal for each alignment according to the Bayesian information criterion in MEGAX [18].

## 3. Results

We tested six specimens with detectable CCHFV (*n* = 4) and JMTV (*n* = 2) genome sequences, as processed ticks (*n* = 4) or cell culture supernatants (*n* = 2) (Table 1). A direct comparison of unbiased NGS and SISPA followed by nanopore sequencing data was possible in four specimens with single (*n* = 2) and pooled (*n* = 2) ticks. Here, higher total or virus-specific read counts were observed in NGS, within a fold range of 10^1^–10^4^ (Table 1). In individual specimens, the ratio of total to specific reads were also lower in SISPA. Meanwhile, SISPA read counts in culture supernatants were significantly elevated in comparison to single or pooled ticks.

Genome segments of both target viruses could be successfully identified and characterized by SISPA (Table 2). In most tick specimens, identities over 99% were noted in all target genome segments. In specimens with single ticks (specimens 1 and 2), the CCHFV M segment sequence data could not be generated or a poor coverage was observed. Relatively lower coverage (below 70%) was also noted for CCHFV S segment and JMTV segment 4. Base calling errors observed as substitutions, insertion/deletions (Indels) and ambiguous bases (R, Y, S, W, K, M, V or N) were identified in tick specimens (Table 2). These errors were more frequent in specimens with lower total or target read counts. The aligned CCHFV and JMTV sequences generated by SISPA + nanopore sequencing are provided in Supplementary Materials Figure S1.

We further performed SISPA to characterize the target viruses in cell culture supernatants. Here, this approach produced better sequence data, with few Indels and no ambiguities. We did not use an alternate method for genome sequencing in these specimens; therefore, the number and rate of possible base calling errors appearing as substitutions could not be determined. However, pairwise comparisons have revealed these viruses to be at least 93% identical to phylogenetically or geographically related viruses. The obtained CCHFV genome showed highest sequence identities with isolate Saf (Figure S1). It also exhibited 93−97% similarities in all genome segments to local viruses and placed within the CCHFV Europe 1 cluster in the maximum-likelihood analyses (Figure S2). The JMTV isolate was obtained from the inoculation of the tick pool used in unbiased NGS and SISPA+ nanopore sequencing. This virus exhibited 95.2–100% identities to local JMTVs in all genome segments and phylogenetically clustered with geographically related viruses (Figure S3).

## 4. Discussion

In this study, we present the findings of an application of the SISPA protocol in combination with nanopore sequencing for the detection of two tick-borne pathogenic viruses with segmented genomes. Although SISPA can be coupled with various NGS platforms, we preferred the third generation nanopore sequencing due to its portability, reduced costs and time for obtaining results. Overall, this strategy generated sufficient sequence output to identify and characterize the target viruses in single or pooled ticks and cell culture supernatants. However, differences in sequence yield, as observed in total and specific read counts, were apparent (Table 1). Moreover, higher quality sequence data could be retrieved from specimens with increased total and specific reads (Table 2). Although a direct comparison between methods was not possible in culture-grown viruses, it is likely that the reduced rate of base calling errors in these specimens was due to higher viral loads. Furthermore, variations in sequence recovery from individual genome segments were noted in JMTV and CCHFV, particularly in the relatively lower coverage of the CCHFV M segment. A point of concern in viral enrichment via SISPA is the inaccuracy or sequence bias that might be introduced by the SISPA in the specimen sequence repertoire. Although this requires in-depth investigation, especially for RNA viruses and in specimens with complex viromes, it is reported to have a minor impact on beta diversity studies of human saliva for DNA viruses [19]. Despite these observations, the SISPA-nanopore sequencing is still superior to screening by PCR, as it enables sequence-independent virus enrichment and produces significant genome sequence data—a major advantage for outbreak investigations, where screening individual agents is expensive and impractical. In contrast to other NGS approaches, nanopore sequencing can be readily performed in resource-limited settings or in field conditions, as demonstrated previously during the Ebola outbreak [20]. An alternative strategy would be the ultra-multiplexed PCR followed by nanopore sequencing, which we have assessed for viral hemorrhagic fever agents previously [21]. Such specific primer-based approaches can be utilized for particular tasks but will be insufficient in highly divergent or novel pathogens, where SISPA enrichment would provide genome-wide pathogen description. 

The current study has several limitations to be addressed. First, we were compelled to use different aliquots for unbiased NGS and SISPA + nanopore sequencing, due to specimen availability. Although the homogenized aliquots employed in the study were kept securely at −80 without freeze/thaws, potential variations affecting quantitative and qualitative sequence output in SISPA experiments cannot be ruled out. Another shortcoming is the lack of standardized specimens with known copy number of viral genomes, evaluated in parallel with the specimens in the experiments. A more informative comparison would be possible using several spiked specimens of identical matrices, as tick pools and culture supernatants, enabling calculations on the limits of detection and optimal performance in the sequencing step. Moreover, the impact of SISPA enrichment for metagenomic virus detection could not be evaluated in this setting. Nevertheless, the study demonstrates a successful proof-of-concept that can produce sufficient sequence data even in a suboptimal setting. We are currently working on developing optimized protocols and pipelines targeting ticks as well as mosquitoes, using well-characterized specimens and several vector-borne viruses. 

In conclusion, the SISPA + nanopore sequencing approach was successful in generating virus genome sequence data from tick specimens and culture-grown tick-borne viruses. It will provide an invaluable tool for broad-range detection of viruses in vectors with further optimization, for which studies are underway.

## Figures and Tables

**Table 1:** Study specimens and assay-based read outputs.

Specimen	Content	Target	Read Count				Ratio	
			NGS		SISPA			
			Total	Specific	Total	Specific	Total	Specific
1	Single tick (*Rhipicephalus turanicus*)	CCHFV	885,295	35,471	84,421	61	10.5	581.5
2	Single tick (*Rhipicephalus sanguineus* s. l.)	CCHFV	1,319,498	14,073	136,582	404	9.7	34.8
3	Pooled ticks (*Rhipicephalus bursa*) 9 individuals	CCHFV	1,009,534	331,006	82,513	2054	12.2	161.2
4	Pooled ticks (*Rhipicephalus bursa*) 15 individuals	JMTV	1,852,008	467,009	33,024	294	56.1	1588.5
5	Cell culture supernatant	CCHFV	*n*.a.	*n*.a.	433,112	1630	*n*.a.	*n*.a.
6	Cell culture supernatant	JMTV	*n*.a.	*n*.a.	111,482	14,407	*n*.a.	*n*.a.

*n*.a.: not available/not applicable.

**Table 2:** Virus and genome-specific comparison of the SISPA sequence data.

Virus	Origin	Genome Segment/Gene	Sequence Length		Coverage	Identity	Indels	Substitutions	Ambiguities	Homopolymers Corrected
			NGS	SISPA						
CCHFV	Specimen 1	L	12,122	7347	60.6%	99.8%	4	2	3	22
		M	5352	−	−	−	−	−	−	
		S	1573	1372	87.2%	99.5%	1	1	5	2
	Specimen 2	L	12,122	11,725	96.7%	99.9%	1	3	7	20
		M	5404	1878	34.7%	99.6%	5	−	3	5
		S	1573	989	62.8%	99.8%	1	−	−	1
	Specimen 3	L	12,122	12,099	99.8%	99.9%	−	2		11
		M	5352	5338	99.7%	99.8%	1	2	1	14
		S	1573	1568	99.6%	100%	−	−	−	1
	Specimen 5 ^a^	L	12,121	12,116	99.9%	*n*.a.	−	*n*.a.	−	11
		M	5332	5314	99.6%	*n*.a.	−	*n*.a.	1	11
		S	1621	1597	98.5%	*n*.a.	−	*n*.a.	1	4
JMTV	Specimen 4	1 (NSP1)	2894	2732	94%	99.9%	1	1	1	6
		2 (VP1)	2549	2087	81.8%	99.8%	−	1	2	8
		3 (NSP2)	2643	2538	96.0%	99.9%	−	2	−	3
		4 (VP2/3)	2635	1547	58.7%	99.2%	4	5	2	4
	Specimen 6 ^b^	1 (NSP1)	2894	2879	99.4%	99.9%	1	0.1	−	11
		2 (VP1)	2549	2514	98.6%	99.8%	−	0.3	−	10
		3 (NSP2)	2643	2610	98.7%	100%	−	−	−	2
		4 (VP2/3)	2635	2592	98.3%	99.8%	−	0.4	−	5

*n*.a.: not available/not applicable; ^a^ no previously-available genome sequence data, coverage and identities are based on the most similar virus genome in GenBank ^b^ as compared to next generation sequencing (NGS) of the initial tick pool.

## Data Availability

All sequences generated by unbiased sequencing were previously submitted to GenBank and assigned accession numbers MN486258, MN486260, MN486265, MN486269, MN811030-MN811037. All remaining data are available in the article supplements.

## Supplementary Materials

The following are available online at https://www.mdpi.com/1999-4915/13/2/203/s1, Figure S1: Alignment of the CCHFV and JMTV sequences generated in the study, using reference genomes from unbiased NGS or viruses with closest identity in public databases. Figure S2: The maximum likelihood analysis of the CCHFV genome in individual segments. The bootstrap consensus trees are constructed using the Tamura-Nei model, gamma distributed with invariant sites (G + I) for 500 replications. The sequences generated in this study are indicated with a symbol and specimen code. Viruses included in the analyses are indicated by GenBank accession number and strain/isolate name. Nairobi sheep disease virus is analyzed as an outgroup. Bootstrap values higher than 50 are shown in the trees. Major CCHFV clades are indicated in the L segment tree. Figure S3: The maximum likelihood analysis of the JMTV genome in individual segments. The trees are constructed using the general time reversible (GTR) model, gamma distributed with invariant sites (G + I) for 500 replications. The sequences generated in this study are indicated with a symbol and specimen code. Viruses included in the analyses are indicated by GenBank accession number and strain/isolate name. Bootstrap values higher than 50 are shown in the trees.
