# Supplementary material for: Utility of a Sequence-Independent, Single-Primer-Amplification (SISPA) and Nanopore Sequencing Approach for Detection and Characterization of Tick-Borne Viral Pathogens
Source: Viruses. 2021 Jan 29;13(2):203. doi: 10.3390/v13020203 (PMC7911436; doi:10.3390/v13020203)
Supplement: Supplementary file 1 [file viruses-13-00203-s001.zip › Figure S2.pdf]

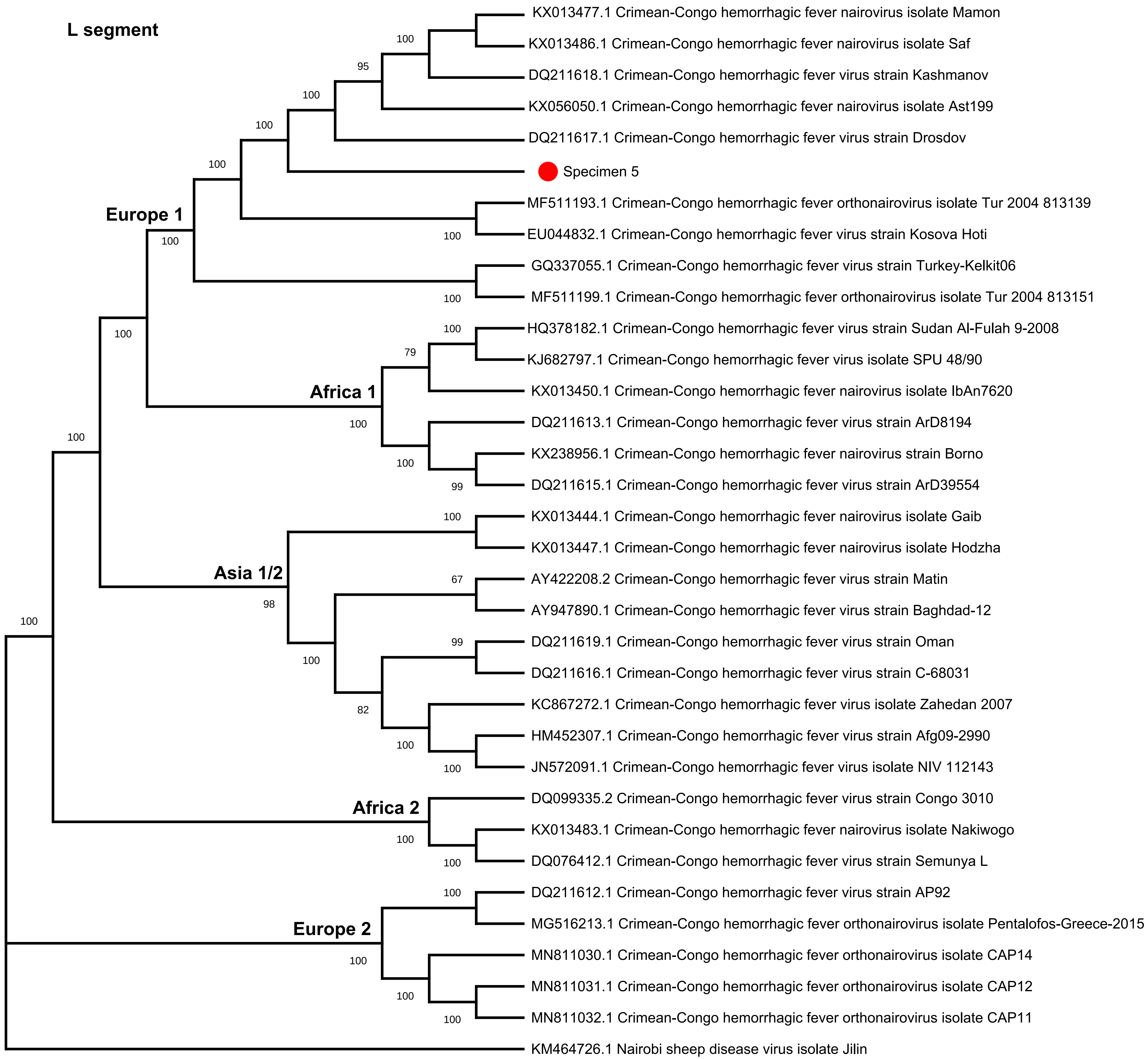

FIGURE S2: The maximum likelihood analysis of the CCHFV genome in individual segments. The bootstrap consensus trees are constructed using the Tamura-Nei model, gamma distributed with invariant sites (G+I) for 500 replications. The sequences generated in this study are indicated with a symbol and specimen code. Viruses included in the analyses are indicated by GenBank accession number and strain/isolate name. Nairobi sheep disease virus is analysed as an outgroup. Bootstrap values higher than 50 are shown in the trees. Major CCHFV clades are indicated in the L segment tree.

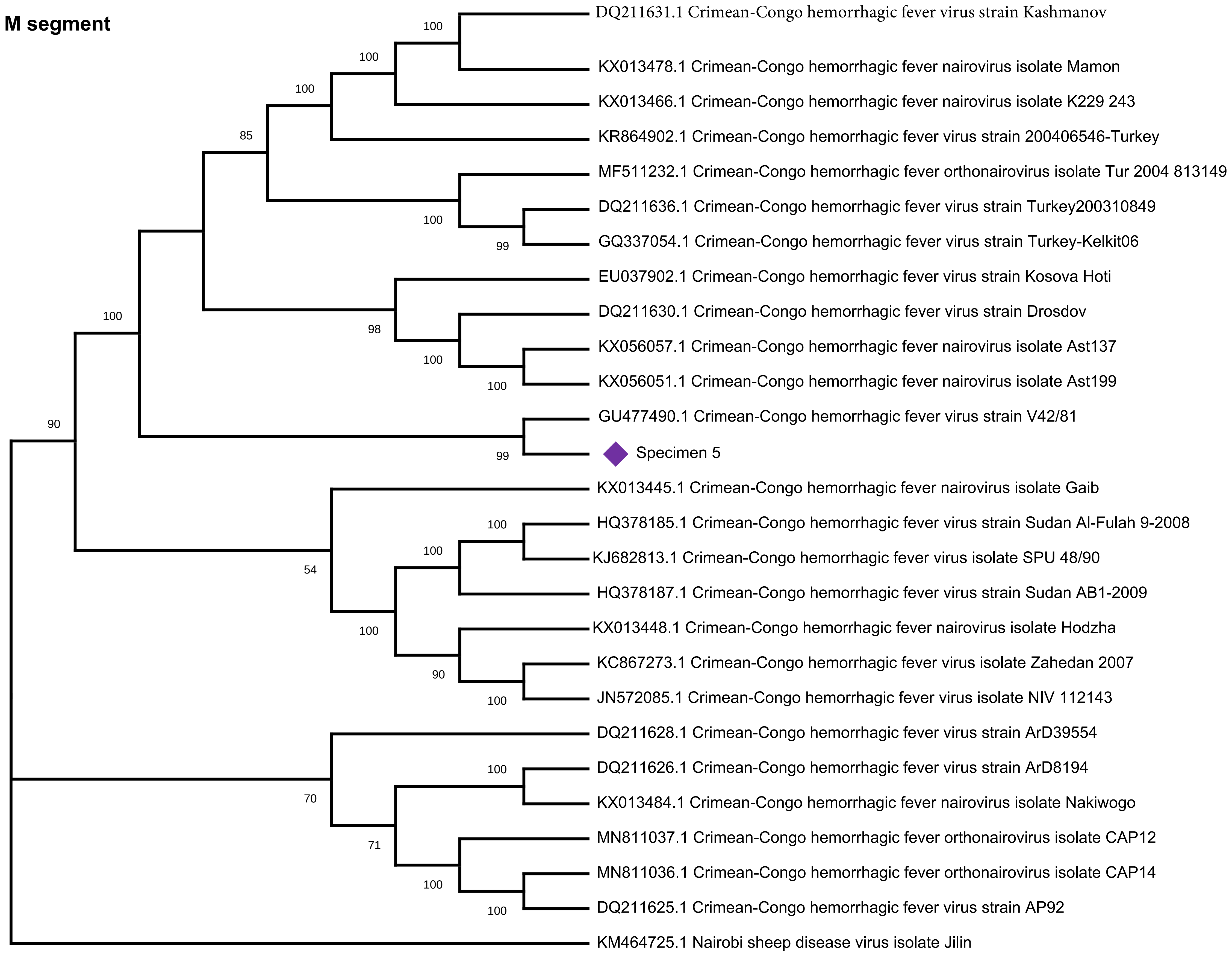

M segment
DQ211631.1 Crimean-Congo hemorrhagic fever virus strain Kashmanov
KX013478.1 Crimean-Congo hemorrhagic fever nairovirus isolate Mamon
KX013466.1 Crimean-Congo hemorrhagic fever nairovirus isolate K229 243
KR864902.1 Crimean-Congo hemorrhagic fever virus strain 200406546-Turkey
MF511232.1 Crimean-Congo hemorrhagic fever orthonairovirus isolate Tur 2004 813149
DQ211636.1 Crimean-Congo hemorrhagic fever virus strain Turkey200310849
GQ337054.1 Crimean-Congo hemorrhagic fever virus strain Turkey-Kelkit06
EU037902.1 Crimean-Congo hemorrhagic fever virus strain Kosova Hoti
DQ211630.1 Crimean-Congo hemorrhagic fever virus strain Drosdov
KX056057.1 Crimean-Congo hemorrhagic fever nairovirus isolate Ast137
KX056051.1 Crimean-Congo hemorrhagic fever nairovirus isolate Ast199
GU477490.1 Crimean-Congo hemorrhagic fever virus strain V42/81
Specimen 5
KX013445.1 Crimean-Congo hemorrhagic fever nairovirus isolate Gaib
HQ378185.1 Crimean-Congo hemorrhagic fever virus strain Sudan Al-Fulah 9-2008
KJ682813.1 Crimean-Congo hemorrhagic fever virus isolate SPU 48/90
HQ378187.1 Crimean-Congo hemorrhagic fever virus strain Sudan AB1-2009
KX013448.1 Crimean-Congo hemorrhagic fever nairovirus isolate Hodzha
KC867273.1 Crimean-Congo hemorrhagic fever virus isolate Zahedan 2007
JN572085.1 Crimean-Congo hemorrhagic fever virus isolate NIV 112143
DQ211628.1 Crimean-Congo hemorrhagic fever virus strain ArD39554
DQ211626.1 Crimean-Congo hemorrhagic fever virus strain ArD8194
KX013484.1 Crimean-Congo hemorrhagic fever nairovirus isolate Nakiwogo
MN811037.1 Crimean-Congo hemorrhagic fever orthonairovirus isolate CAP12
MN811036.1 Crimean-Congo hemorrhagic fever orthonairovirus isolate CAP14
DQ211625.1 Crimean-Congo hemorrhagic fever virus strain AP92
KM464725.1 Nairobi sheep disease virus isolate Jilin

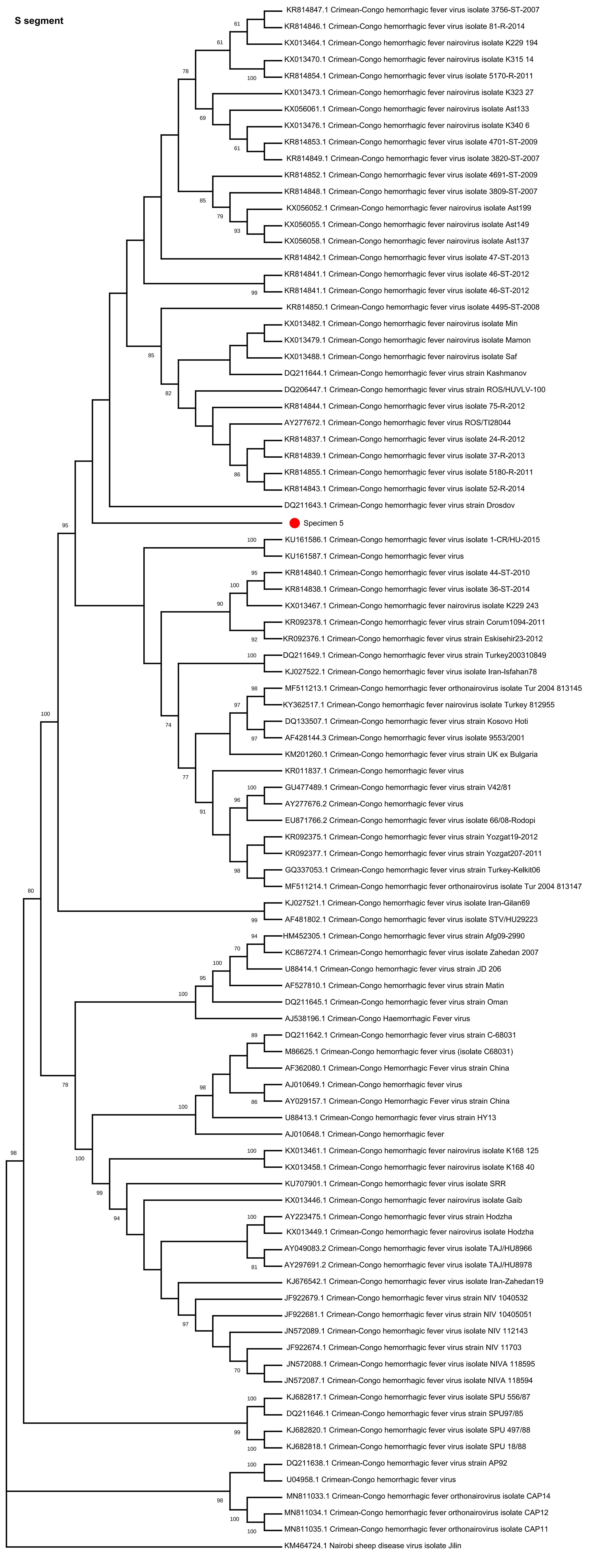
S segment
KR814847.1 Crimean-Congo hemorrhagic fever virus isolate 3756-ST-2007
KR814846.1 Crimean-Congo hemorrhagic fever virus isolate 81-R-2014
KX013464.1 Crimean-Congo hemorrhagic fever nairovirus isolate K229 194
KX013470.1 Crimean-Congo hemorrhagic fever nairovirus isolate K315 14
KR814854.1 Crimean-Congo hemorrhagic fever virus isolate 5170-R-2011
KX013473.1 Crimean-Congo hemorrhagic fever nairovirus isolate K323 27
KX056061.1 Crimean-Congo hemorrhagic fever nairovirus isolate Ast133
KX013476.1 Crimean-Congo hemorrhagic fever nairovirus isolate K340 6
KR814853.1 Crimean-Congo hemorrhagic fever virus isolate 4701-ST-2009
KR814849.1 Crimean-Congo hemorrhagic fever virus isolate 3820-ST-2007
KR814852.1 Crimean-Congo hemorrhagic fever virus isolate 4691-ST-2009
KR814848.1 Crimean-Congo hemorrhagic fever virus isolate 3809-ST-2007
KX056052.1 Crimean-Congo hemorrhagic fever nairovirus isolate Ast199
KX056055.1 Crimean-Congo hemorrhagic fever nairovirus isolate Ast149
KX056058.1 Crimean-Congo hemorrhagic fever nairovirus isolate Ast137
KR814842.1 Crimean-Congo hemorrhagic fever virus isolate 47-ST-2013
KR814841.1 Crimean-Congo hemorrhagic fever virus isolate 46-ST-2012
KR814841.1 Crimean-Congo hemorrhagic fever virus isolate 46-ST-2012
KR814850.1 Crimean-Congo hemorrhagic fever virus isolate 4495-ST-2008
KX013482.1 Crimean-Congo hemorrhagic fever nairovirus isolate Min
KX013479.1 Crimean-Congo hemorrhagic fever nairovirus isolate Mamon
KX013488.1 Crimean-Congo hemorrhagic fever nairovirus isolate Saf
DQ211644.1 Crimean-Congo hemorrhagic fever virus strain Kashmanov
DQ206447.1 Crimean-Congo hemorrhagic fever virus strain ROS/HUVLV-100
KR814844.1 Crimean-Congo hemorrhagic fever virus isolate 75-R-2012
AY277672.1 Crimean-Congo hemorrhagic fever virus ROS/TI28044
KR814837.1 Crimean-Congo hemorrhagic fever virus isolate 24-R-2012
KR814839.1 Crimean-Congo hemorrhagic fever virus isolate 37-R-2013
KR814855.1 Crimean-Congo hemorrhagic fever virus isolate 5180-R-2011
KR814843.1 Crimean-Congo hemorrhagic fever virus isolate 52-R-2014
DQ211643.1 Crimean-Congo hemorrhagic fever virus strain Drosdov
Specimen 5
KU161586.1 Crimean-Congo hemorrhagic fever virus isolate 1-CR/HU-2015
KU161587.1 Crimean-Congo hemorrhagic fever virus
KR814840.1 Crimean-Congo hemorrhagic fever virus isolate 44-ST-2010
KR814838.1 Crimean-Congo hemorrhagic fever virus isolate 36-ST-2014
KX013467.1 Crimean-Congo hemorrhagic fever nairovirus isolate K229 243
KR092378.1 Crimean-Congo hemorrhagic fever virus strain Corum1094-2011
KR092376.1 Crimean-Congo hemorrhagic fever virus strain Eskisehir23-2012
DQ211649.1 Crimean-Congo hemorrhagic fever virus strain Turkey200310849
KJ027522.1 Crimean-Congo hemorrhagic fever virus isolate Iran-Isfahan78
MF511213.1 Crimean-Congo hemorrhagic fever orthonairovirus isolate Tur 2004 813145
KY362517.1 Crimean-Congo hemorrhagic fever nairovirus isolate Turkey 812955
DQ133507.1 Crimean-Congo hemorrhagic fever virus strain Kosovo Hoti
AF428144.3 Crimean-Congo hemorrhagic fever virus isolate 9553/2001
KM201260.1 Crimean-Congo hemorrhagic fever virus strain UK ex Bulgaria
KR011837.1 Crimean-Congo hemorrhagic fever virus
GU477489.1 Crimean-Congo hemorrhagic fever virus strain V42/81
AY277676.2 Crimean-Congo hemorrhagic fever virus
EU871766.2 Crimean-Congo hemorrhagic fever virus isolate 66/08-Rodopi
KR092375.1 Crimean-Congo hemorrhagic fever virus strain Yozgat19-2012
KR092377.1 Crimean-Congo hemorrhagic fever virus strain Yozgat207-2011
GQ337053.1 Crimean-Congo hemorrhagic fever virus strain Turkey-Kelkit06
MF511214.1 Crimean-Congo hemorrhagic fever orthonairovirus isolate Tur 2004 813147
KJ027521.1 Crimean-Congo hemorrhagic fever virus isolate Iran-Gilan69
AF481802.1 Crimean-Congo hemorrhagic fever virus isolate STV/HU29223
HM452305.1 Crimean-Congo hemorrhagic fever virus strain Afg09-2990
KC867274.1 Crimean-Congo hemorrhagic fever virus isolate Zahedan 2007
U88414.1 Crimean-Congo hemorrhagic fever virus strain JD 206
AF527810.1 Crimean-Congo hemorrhagic fever virus strain Matin
DQ211645.1 Crimean-Congo hemorrhagic fever virus strain Oman
AJ538196.1 Crimean-Congo Haemorrhagic Fever virus
DQ211642.1 Crimean-Congo hemorrhagic fever virus strain C-68031
M86625.1 Crimean-Congo hemorrhagic fever virus (isolate C68031)
AF362080.1 Crimean-Congo Hemorrhagic Fever virus strain China
AJ010649.1 Crimean-Congo hemorrhagic fever virus
AY029157.1 Crimean-Congo Hemorrhagic Fever virus strain China
U88413.1 Crimean-Congo hemorrhagic fever virus strain HY13
AJ010648.1 Crimean-Congo hemorrhagic fever
KX013461.1 Crimean-Congo hemorrhagic fever nairovirus isolate K168 125
KX013458.1 Crimean-Congo hemorrhagic fever nairovirus isolate K168 40
KU707901.1 Crimean-Congo hemorrhagic fever virus isolate SRR
KX013446.1 Crimean-Congo hemorrhagic fever nairovirus isolate Gaib
AY223475.1 Crimean-Congo hemorrhagic fever virus strain Hodzha
KX013449.1 Crimean-Congo hemorrhagic fever nairovirus isolate Hodzha
AY049083.2 Crimean-Congo hemorrhagic fever virus isolate TAJ/HU8966
AY297691.2 Crimean-Congo hemorrhagic fever virus isolate TAJ/HU8978
KJ676542.1 Crimean-Congo hemorrhagic fever virus isolate Iran-Zahedan19
JF922679.1 Crimean-Congo hemorrhagic fever virus strain NIV 1040532
JF922681.1 Crimean-Congo hemorrhagic fever virus strain NIV 10405051
JN572089.1 Crimean-Congo hemorrhagic fever virus isolate NIV 112143
JF922674.1 Crimean-Congo hemorrhagic fever virus strain NIV 11703
JN572088.1 Crimean-Congo hemorrhagic fever virus isolate NIVA 118595
JN572087.1 Crimean-Congo hemorrhagic fever virus isolate NIVA 118594
KJ682817.1 Crimean-Congo hemorrhagic fever virus isolate SPU 556/87
DQ211646.1 Crimean-Congo hemorrhagic fever virus strain SPU97/85
KJ682820.1 Crimean-Congo hemorrhagic fever virus isolate SPU 497/88
KJ682818.1 Crimean-Congo hemorrhagic fever virus isolate SPU 18/88
DQ211638.1 Crimean-Congo hemorrhagic fever virus strain AP92
U04958.1 Crimean-Congo hemorrhagic fever virus
MN811033.1 Crimean-Congo hemorrhagic fever orthonairovirus isolate CAP14
MN811034.1 Crimean-Congo hemorrhagic fever orthonairovirus isolate CAP12
MN811035.1 Crimean-Congo hemorrhagic fever orthonairovirus isolate CAP11
KM464724.1 Nairobi sheep disease virus isolate Jilin
