# Supplementary material for: Utility of a Sequence-Independent, Single-Primer-Amplification (SISPA) and Nanopore Sequencing Approach for Detection and Characterization of Tick-Borne Viral Pathogens
Source: Viruses. 2021 Jan 29;13(2):203. doi: 10.3390/v13020203 (PMC7911436; doi:10.3390/v13020203)
Supplement: Supplementary file 1 [file viruses-13-00203-s001.zip › Figure S3.pdf]

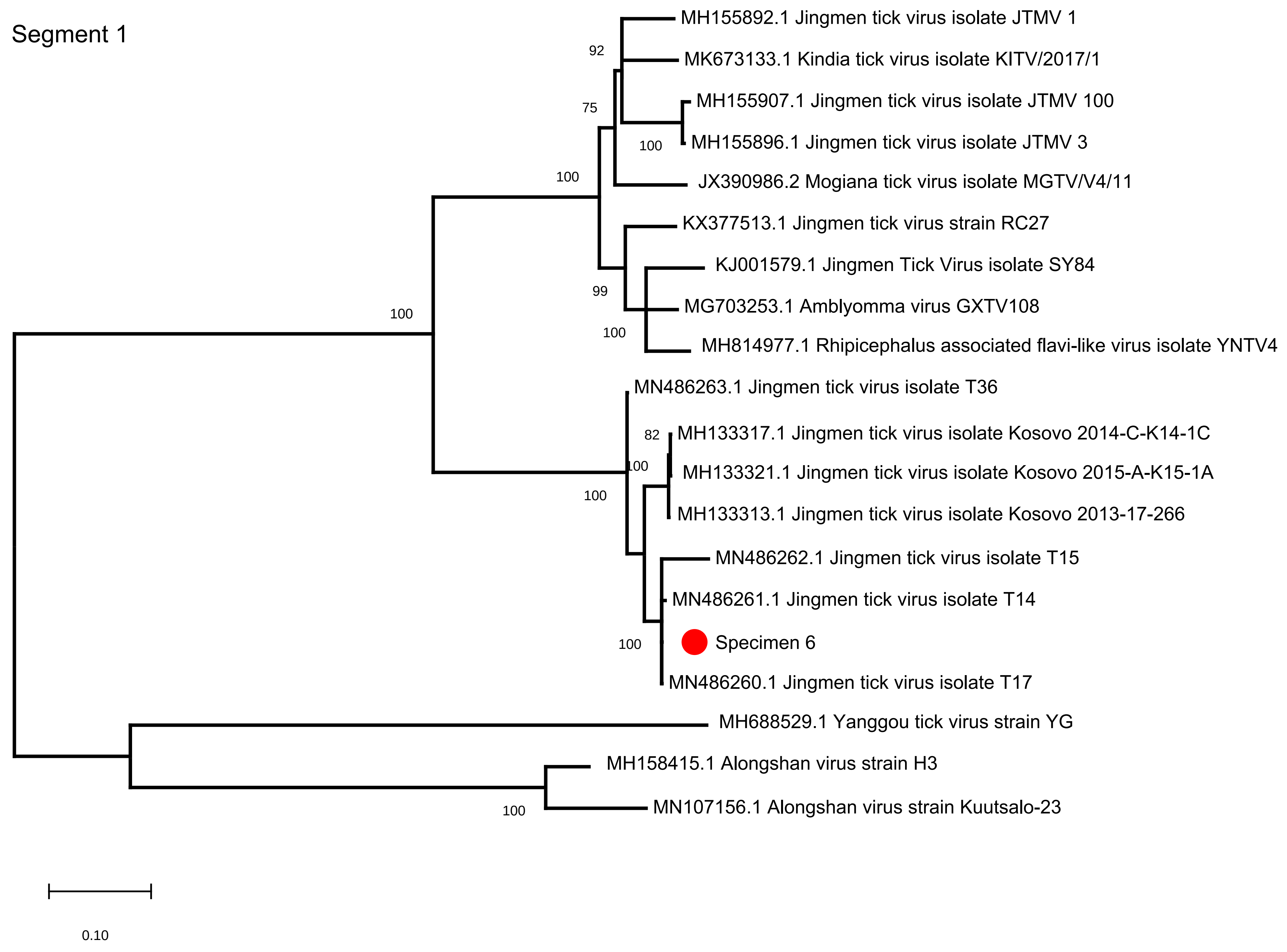

FIGURE S: The maximum likelihood analysis of the JMTV sequences in individual genomic segments. The trees are constructed using the general time reversible (GTR) model, gamma distributed with invariant sites (G+I) for 500 replications. The sequences generated in this study are indicated with a symbol and specimen code. Viruses included in the analyses are indicated by GenBank accession number and strain/isolate name. Bootstrap values higher than 50 are shown in the trees.

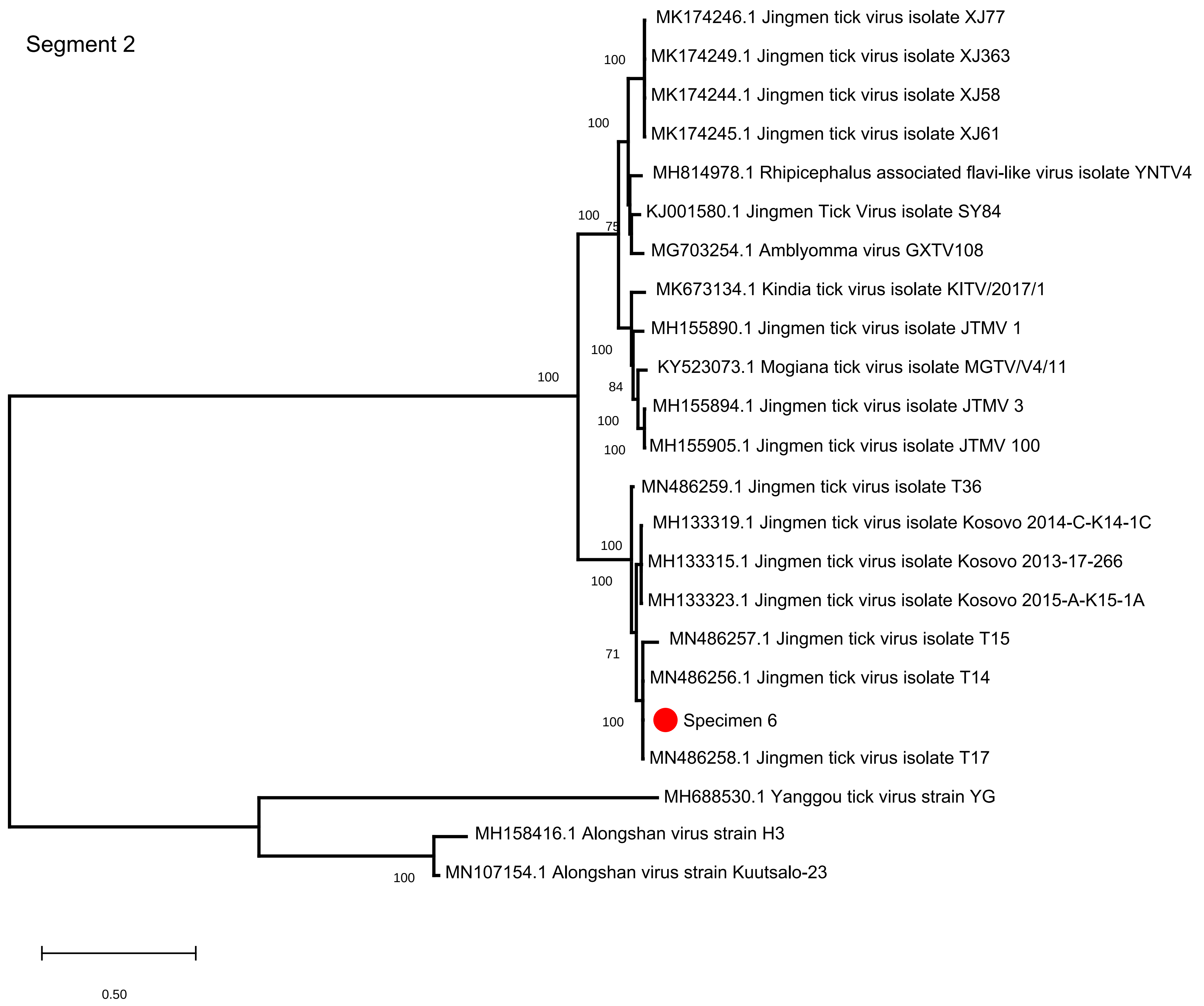
Segment 2
MK174246.1 Jingmen tick virus isolate XJ77
MK174249.1 Jingmen tick virus isolate XJ363
MK174244.1 Jingmen tick virus isolate XJ58
MK174245.1 Jingmen tick virus isolate XJ61
MH814978.1 Rhipicephalus associated flavi-like virus isolate YNTV4
KJ001580.1 Jingmen Tick Virus isolate SY84
MG703254.1 Amblyomma virus GXTV108
MK673134.1 Kindia tick virus isolate KITV/2017/1
MH155890.1 Jingmen tick virus isolate JTMV 1
KY523073.1 Mogiana tick virus isolate MGTV/V4/11
MH155894.1 Jingmen tick virus isolate JTMV 3
MH155905.1 Jingmen tick virus isolate JTMV 100
MN486259.1 Jingmen tick virus isolate T36
MH133319.1 Jingmen tick virus isolate Kosovo 2014-C-K14-1C
MH133315.1 Jingmen tick virus isolate Kosovo 2013-17-266
MH133323.1 Jingmen tick virus isolate Kosovo 2015-A-K15-1A
MN486257.1 Jingmen tick virus isolate T15
MN486256.1 Jingmen tick virus isolate T14
Specimen 6
MN486258.1 Jingmen tick virus isolate T17
MH688530.1 Yanggou tick virus strain YG
MH158416.1 Alongshan virus strain H3
MN107154.1 Alongshan virus strain Kuutsalo-23
100
100
100
75
100
100
84
100
100
100
100
71
100
100
100
0.50

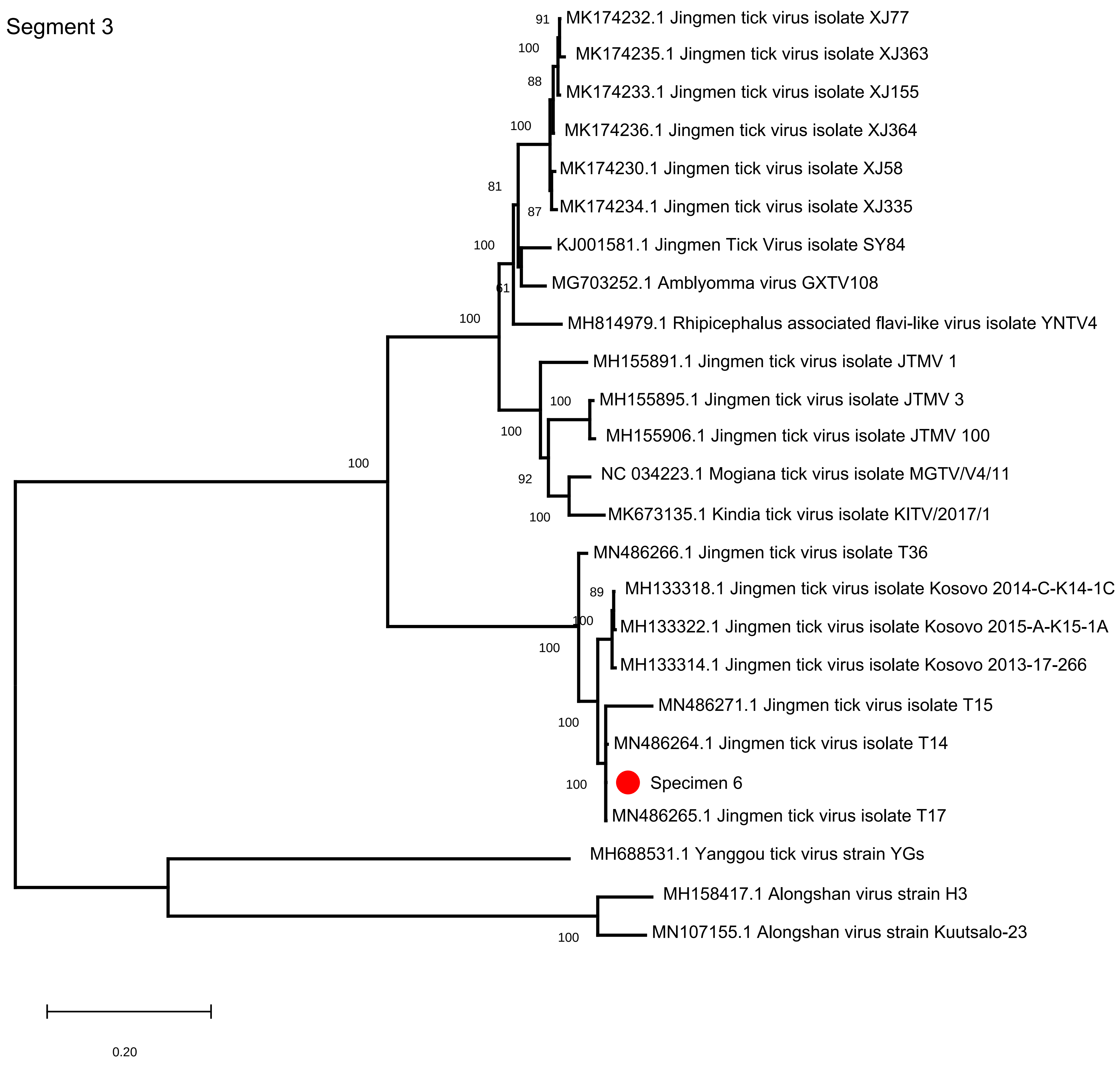

Segment 3
MK174232.1 Jingmen tick virus isolate XJ77
MK174235.1 Jingmen tick virus isolate XJ363
MK174233.1 Jingmen tick virus isolate XJ155
MK174236.1 Jingmen tick virus isolate XJ364
MK174230.1 Jingmen tick virus isolate XJ58
MK174234.1 Jingmen tick virus isolate XJ335
KJ001581.1 Jingmen Tick Virus isolate SY84
MG703252.1 Amblyomma virus GXTV108
MH814979.1 Rhipicephalus associated flavi-like virus isolate YNTV4
MH155891.1 Jingmen tick virus isolate JTMV 1
MH155895.1 Jingmen tick virus isolate JTMV 3
MH155906.1 Jingmen tick virus isolate JTMV 100
NC 034223.1 Mogiana tick virus isolate MGTV/V4/11
MK673135.1 Kindia tick virus isolate KITV/2017/1
MN486266.1 Jingmen tick virus isolate T36
MH133318.1 Jingmen tick virus isolate Kosovo 2014-C-K14-1C
MH133322.1 Jingmen tick virus isolate Kosovo 2015-A-K15-1A
MH133314.1 Jingmen tick virus isolate Kosovo 2013-17-266
MN486271.1 Jingmen tick virus isolate T15
MN486264.1 Jingmen tick virus isolate T14
Specimen 6
MN486265.1 Jingmen tick virus isolate T17
MH688531.1 Yanggou tick virus strain YGs
MH158417.1 Alongshan virus strain H3
MN107155.1 Alongshan virus strain Kuutsalo-23
91
100
88
100
81
87
100
61
100
100
100
100
92
100
100
89
100
100
100
100
100
0.20

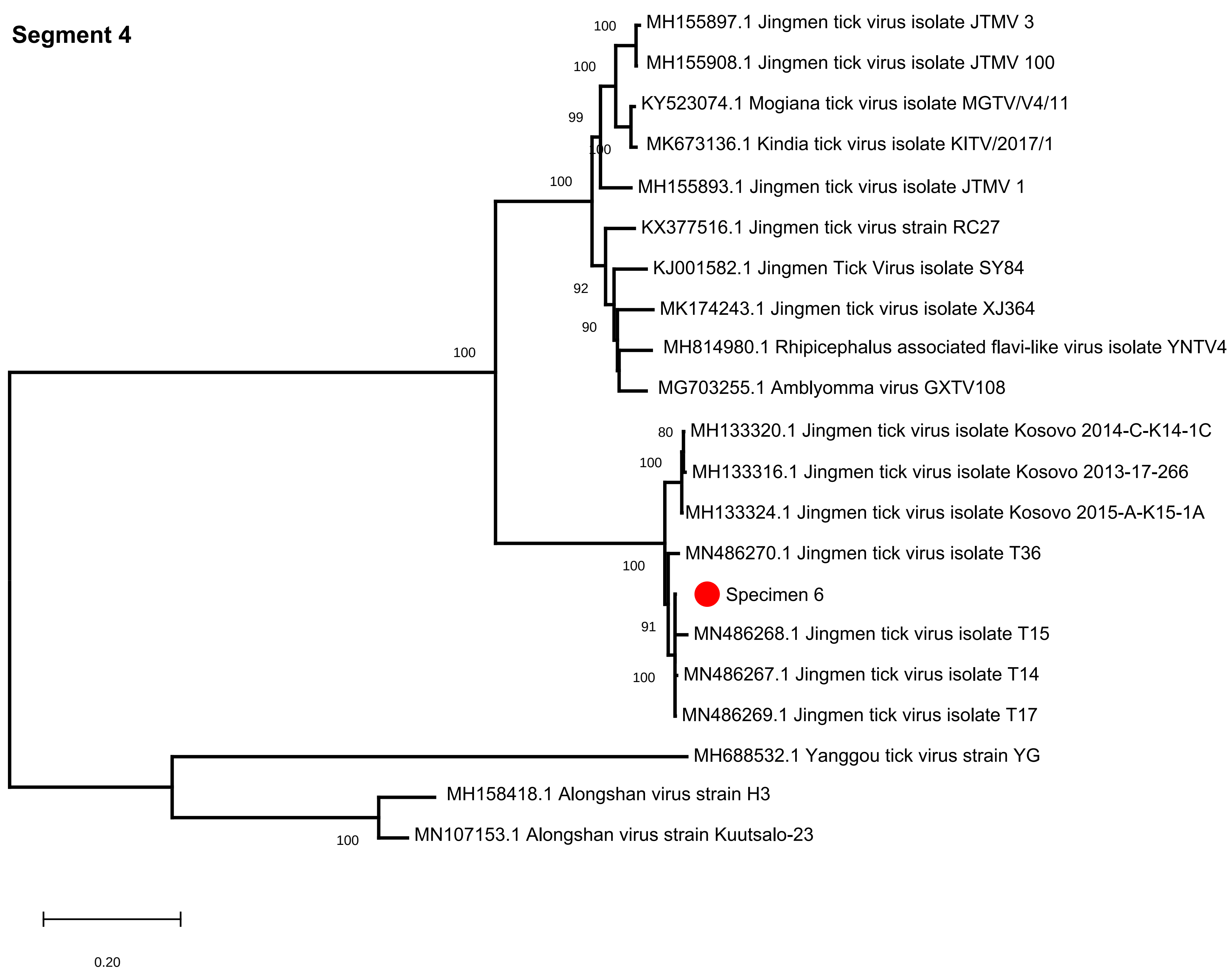

Segment 4
MH155897.1 Jingmen tick virus isolate JTMV 3
MH155908.1 Jingmen tick virus isolate JTMV 100
KY523074.1 Mogiana tick virus isolate MGTV/V4/11
MK673136.1 Kindia tick virus isolate KITV/2017/1
MH155893.1 Jingmen tick virus isolate JTMV 1
KX377516.1 Jingmen tick virus strain RC27
KJ001582.1 Jingmen Tick Virus isolate SY84
MK174243.1 Jingmen tick virus isolate XJ364
MH814980.1 Rhipicephalus associated flavi-like virus isolate YNTV4
MG703255.1 Amblyomma virus GXTV108
MH133320.1 Jingmen tick virus isolate Kosovo 2014-C-K14-1C
MH133316.1 Jingmen tick virus isolate Kosovo 2013-17-266
MH133324.1 Jingmen tick virus isolate Kosovo 2015-A-K15-1A
MN486270.1 Jingmen tick virus isolate T36
Specimen 6
MN486268.1 Jingmen tick virus isolate T15
MN486267.1 Jingmen tick virus isolate T14
MN486269.1 Jingmen tick virus isolate T17
MH688532.1 Yanggou tick virus strain YG
MH158418.1 Alongshan virus strain H3
MN107153.1 Alongshan virus strain Kuutsalo-23
100
100
99
100
100
92
90
100
80
100
100
91
100
100
100
0.20
